# Functional Bladder Paraganglioma as an Incidental Finding During Infertility Workup

**DOI:** 10.7759/cureus.18815

**Published:** 2021-10-16

**Authors:** Fouad Hajji, Abderrazak Benazzouz, Nabil Hammoune, Mohamed Amine Azami, Omar Ghoundale

**Affiliations:** 1 Department of Urology, Ibn Sina Military Hospital, Marrakech, MAR; 2 Department of Radiology, Ibn Sina Military Hospital, Marrakech, MAR; 3 Department of Pathology, Ibn Sina Military Hospital, Marrakech, MAR

**Keywords:** cystoscopy, laparoscopic partial cystectomy, transvaginal ultrasound, infertility, urinary bladder, paraganglioma

## Abstract

Bladder paragangliomas (PGLs) are extremely rare catecholamine-producing neuroendocrine tumors. They arise more frequently in the trigone and have unpredictable depth and behavior. Most cases typically present with a well-defined set of symptoms triggered by micturition or bladder overdistension. Besides long-term follow-up, they are usually managed by either transurethral resection (TUR) or partial cystectomy (PC). However, about 25% of all documented cases do not manifest clinically, raising both diagnosis and management challenges. This report describes an unusual case of a misdiagnosed, functional PGL arising on the bladder dome, which was fortuitously detected in a 21-year-old female during a fertility workup. Owing to its hypervascular nature and submucosal location, bladder PGL was suspected on ultrasound and CT findings and successfully diagnosed before surgery through biochemical confirmation. It was managed by cystoscopy-guided laparoscopic partial cystectomy (LPC) with good oncological and urinary outcomes, as well as no compromise of her fertility potential. To our best knowledge, this is the first case to be incidentally detected on transvaginal ultrasound during evaluation for infertility. This case also stresses the importance of considering PGL in the differential diagnosis of atypical bladder tumors, as well as conservative management through simultaneous laparoscopy and cystoscopy, when approaching young patients with large functional PGL.

## Introduction

Bladder paragangliomas (PGLs) are very rare neuroendocrine tumors. Since the description of the first case in 1953, its incidence is less than 0.05% of all bladder tumors and less than 1% of all pheochromocytomas [1,2]. Among all genitourinary PGLs, the bladder is usually involved (79.2%) [1]. This catecholamine-producing neoplasm arises from the ganglion cell in the bladder wall, most commonly on the trigone, with an average tumor size of 3.9 cm [1,2]. They have uncertain malignant potential with unpredictable depth and behavior, demonstrating extravesical invasion in up to 50% of cases. How this entity can come about remains unclear, but it may have an inherited genetic susceptibility in up to 50% of cases [3]. It occurs in all age groups but more often in patients aged 20-40 years with no sex predominance [1,2]. Most cases present with a well-defined set of symptoms related to excessive catecholamine release during bladder overdistension and/or micturition, including hypertensive crises, headache, palpitations, sweating, hot flushes that may be accompanied by painless hematuria, post micturition hypotension, or syncope [1-3]. A high index of clinical suspicion should lead to a measurement of 24-hour urine and/or plasma fractionated catecholamines and metanephrines. However, 25% of all documented cases do not manifest clinically [4], incidentally imaged in patients who are evaluated for lower urinary tract symptoms (LUTS) or intermittent painless hematuria, and often recognized intraoperatively during transurethral resection (TUR) or as a histological surprise [5]. This report describes an unusual case of a large, hormonally active primary bladder PGL arising on the bladder dome, initially misdiagnosed but incidentally imaged during a routine infertility workup.

## Case presentation

**Figure 1:**
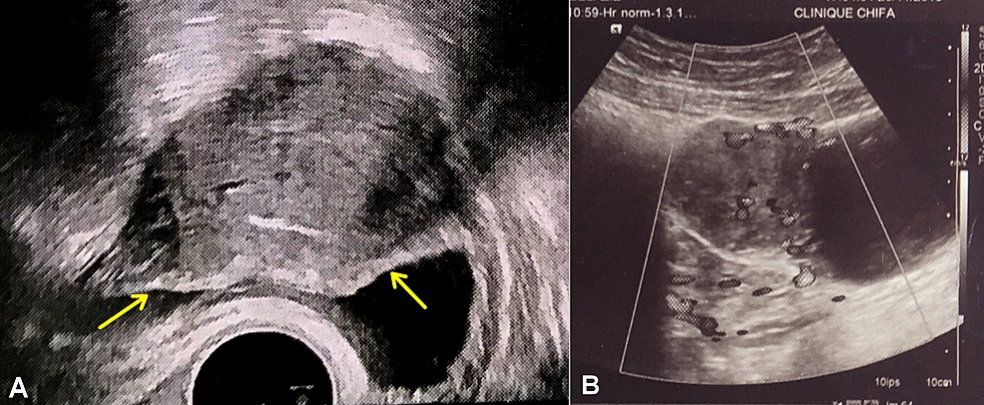
Ultrasound image of the urinary bladder showing an intramural mass along the dome of the bladder. (A) Axial TVS image showing a preserved epithelial lining (yellow arrows). (B) Sagittal transabdominal US image showing marked intralesional and peripheral flow signals on color Doppler.

A 21-year-old female, who has been trying to get pregnant for over two years, was referred by her gynecologist to our urology outpatient clinic because of incidental transvaginal scan (TVS) finding of a bladder intramural mass during routine infertility workup (Figure 1A).

She was a nonsmoker and not exposed to any solvents or chemicals. She denied any history of gross hematuria, LUTS or pelvic pain, weight loss, or appetite disturbance. Nine months prior, she had consulted her general practitioner for intermittent hypertensive attacks during micturition accompanied by headache, sweating, nausea, fatigue, and palpitations of 12 months’ duration. Prior to this clinical scenario, she described herself as healthy with unknown comorbidities. Her doctor had prescribed a calcium channel blocker (10 mg/day of amlodipine) and referred her to a cardiologist for a further workup to assess the potential causes of secondary hypertension. However, she did not show up as she had felt better.

On admission, she had an uncontrolled hypertension of 139/100 mmHg and a heart rate of 82 bpm with no fever. Her physical examination was unremarkable. Transabdominal ultrasound revealed a 7-cm solid mass along the dome and the right lateral wall of the urinary bladder, with hypervascularity on color Doppler (Figure 1B). Urinalysis did not show microscopic hematuria, culture was normal, and cytology was negative for malignancy. Her routine blood tests were otherwise normal.

**Figure 2:**
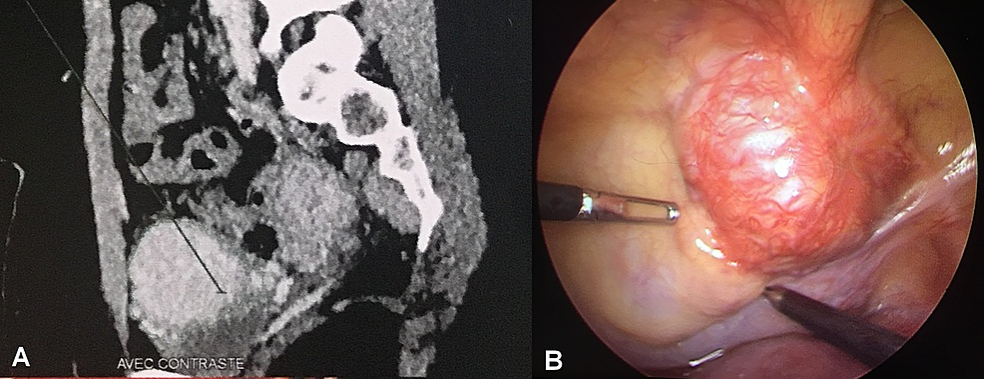
Radiomorphological and intraoperative image showing a highly vascular tumor along the dome of the urinary bladder. (A) Sagittal contrast-enhanced CT image of the abdomen. There were no signs of local invasion and regional lymph node or distant metastasis. The upper urinary tract was normal. (B) Intraoperative laparoscopic view image.

Chest-abdomen-pelvis CT scan demonstrated a localized bladder mass of 70.7 x 33.2 x 47.3 mm along the dome of the urinary bladder, exhibiting significant homogeneous post contrast enhancement (Figure 2A). Given the patient’s medical history, bladder PGL was suspected. Subsequent endocrine testing revealed elevated levels of 24-hour urine metanephrines: total normetanephrine of 4300 mcg (normal < 380) and metanephrine of 220 mcg (normal < 200). Further metaiodobenzylguanidine (MIBG) whole-body scan showed no abnormal tracer-avid lesions. A diagnosis of bladder PGL was considered, albeit with no family history of pheochromocytomas, other related diseases, and unexplained or incompletely explained sudden death.

The patient consented to undergo laparoscopic partial cystectomy (LPC) and subsequently switched on oral alpha-blocker agent for 15 days before, along with high-salt diet and increased water intake. Under general anesthesia and invasive blood pressure monitoring, she was placed in Trendelenburg modified lithotomy position to allow simultaneous cystoscopy, which initially revealed a yellow submucosal mass on the dome and right lateral wall of the bladder. Given the tumor’s location, a ureteral catheter was inserted on the right side. With a transperitoneal approach, laparoscopy showed hypervascular mass on the bladder dome (Figure 2B).

**Figure 3:**
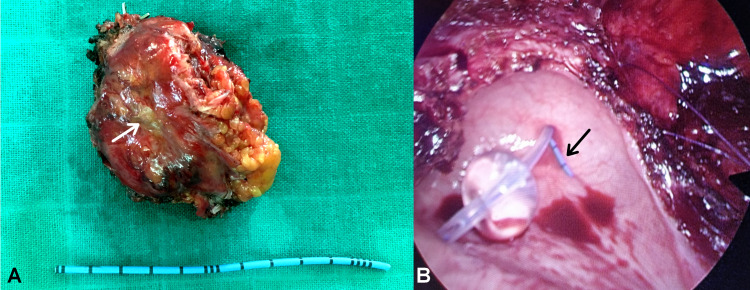
Macroscopic and intraoperative image showing complete resection of the bladder mass. (A) Gross specimen of the bladder mass showing a yellowish tumor measuring 7 x 5 cm, with a macroscopic appearance similar to that of an adrenal tissue (white arrow). (B) Intraoperative laparoscopic view showing sufficient bladder tissue for primary closure. A ureteral catheter was initially inserted in the right side (black arrow).

**Figure 4:**
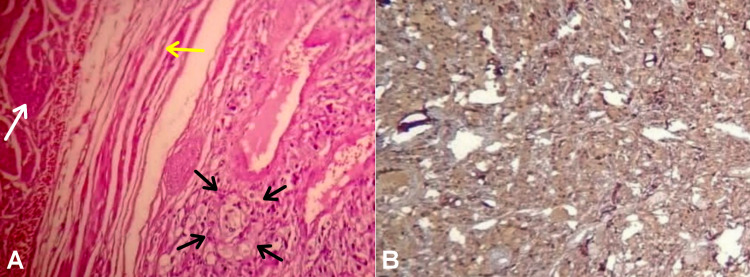
Photomicrograph image showing histological features pathognomonic of paraganglioma. (A) Hematoxylin and eosin staining showing a characteristic Zellballen pattern with polygonal epithelioid cells of abundant eosinophilic cytoplasm arranged in nests and divided by delicate vascular stroma (black arrows). The nests were close to the detrusor muscle bundles (white arrow) but without capsular invasion (yellow arrow) (magnification, x400). (B) Immunohistochemistry for chromogranin showing positive cytoplasmic staining in the tumor cells (magnification, x200).

The specimen was then dissected extravesically under simultaneous cystoscopic guidance and resected completely (Figure 3A), with sufficient bladder tissue for primary closure (Figure 3B). The peri- and postoperative courses were uneventful. Histopathology confirmed the diagnosis of bladder pheochromocytoma, which was staged as functional stage 2 PGL (T2Nx) with negative surgical margins (Figure 4).

The ureteral catheter was removed after two days, and the patient was discharged with urinary catheter removal scheduled on the 10th day. One month later, she become normotensive and had normal levels of urine metanephrines. She was told that her tumor is most likely sporadic and benign; nonetheless, both genetic testing and long-term follow-up were strongly advised. However, she did refuse the genetic screening, concerned about its impact on her relatives and pregnancy planning.

At six months, she denied any hypertensive attacks or voiding difficulties, with no residual tumor on CT scan. At her three-year follow-up, she achieved successful natural pregnancy and had no evidence of local recurrence, distant metastasis, or multifocal disease.

## Discussion

Bladder PGLs may be fortuitously discovered by pelvic ultrasound, CT, and magnetic resonance imaging (MRI) [6]. To our best knowledge, this is the first case of its kind to be incidentally detected on transvaginal ultrasound during infertility workup and managed by cystoscopy-assisted LPC [7,8]. Unlike most common bladder tumors, PGLs are highly vascular neoplasm [3]. Consequently, imaging studies may play an important role in maintaining a high index of suspicion, even in nonfunctional and/or asymptomatic cases. Color Doppler ultrasound and contrast-enhanced CT may demonstrate the vascular nature of these tumors, which was also noticed in our case. On MRI, which also provides knowledge on bladder wall infiltration, these tumors exhibit high-signal intensity on T2-weighted (T2W) images [2,6]. Some PGLs exhibit uptake of radioactive isotopes; therefore, nuclear imaging such as MIBG and positron emission tomography (PET) scans may be useful as functional tools in localizing the tumor [2-4,6]. As these tracers have physiological accumulation in the urinary bladder, the use of an MIBG scan was then helpful in primarily ruling out multifocal and/or metastatic disease in this case.

The endoscopic appearance of bladder PGLs is helpful when endocrine testing or imaging is inconclusive or when TUR of bladder tumor (TURBT) was done incidentally. On cystoscopy, PGL should be considered in the presence of yellowish submucosal mass, especially if the blood pressure shows fluctuations during the procedure [2-4]. However, performing a biopsy of bladder lesions suspicious for PGLs in the outpatient setting is not recommended given the risks of easy bleeding, false-negative results, and labile hypertension.

Pathologically, a PGL consists of polygonal cells harboring abundant cytoplasm and surrounded by a prominent vascular network, with a pathognomonic nested “Zellballen” pattern and staining positive for chromogranin, synaptophysin, and S100 but negative for cytokeratin [2,3,5]. However, there were no reliable histological criteria to rule out malignancy [2,3,9], especially in the current case without metastases.

Currently, more than 50% of all PGLs and 35% of apparently sporadic cases occur in the setting of inherited susceptibility mutations involved in several pheochromocytoma-associated syndromes. It includes multiple endocrine neoplasia type 2, von Hippel-Lindau disease, neurofibromatosis type 1, and hereditary pheochromocytoma-paraganglioma syndromes [3,9]. Hence, a clinically oriented genetic testing should have been performed in this case, especially with an early-onset extra-adrenal pheochromocytoma.

Had PGL not been incidentally imaged, preoperatively diagnosed, and successfully treated, our patient would have been at risk of lethal paroxysm, cardiac disorders, and malignant potential [3,9].

There are currently no guidelines on the surgical management of bladder PGL. Treatment changes according to the tumor’s functionality, size, location, and invasiveness. It can include TUR of bladder tumor (TURBT) and partial and total cystectomy [1-4,6]. The current case could have been managed endoscopically, but the potential risk factors of TURBT include dangerous catecholamine release, bleeding, pathological understaging, and recurrence. TURBT may be feasible, safe, and effective in intramural, nonfunctional tumors smaller than 3 cm, especially if visually complete resection is achieved [2,3]. Consequently, PC is a good treatment option in this condition, especially in young patients highly motivated for regular follow-ups [3]. However, cases with noncontributory radiological and/or hormonal findings, unlike the current patient, should undergo TUR biopsy with histopathological diagnosis of PGL as a safer approach before PC. 

In this case, unlike TURBT, the laparoscopic approach allowed less disruption and en bloc excision of the tumor, resulting in lower intraoperative morbidity and better disease assessment and control [3]. Simultaneous cystoscopy helped in marking initial cystostomy, avoiding injury of the pre-stented right ureter, and planning safety margins. During the laparoscopic excision of the tumor, safe surgical margins may be actually ensured by the help of cystoscopic transillumination [10], cystoscopic tattooing by methylene blue injection or monopolar electrocautery around the tumor [11], or direct visual control as in the current case. Hence, simultaneous cystoscopy seemed easy, safe, and effective as it did not add to increased operative time or morbidity.

## Conclusions

Incidental TVS finding of a bladder PGL during fertility workup is uncommon, yet it should be considered in the differential diagnosis of atypical highly vascular and/or submucosal bladder tumors. Endoscopic management can be used in selected cases. Nevertheless, LPC is the standard treatment option in this condition. This case also favors the notion that a hybrid approach using simultaneous cystoscopy may be superior to pure laparoscopy, providing efficient, secure, and effective procedure, when approaching young patients with large functional PGL on the dome of the urinary bladder.
